# Mass Spectrometric Analysis of Cerebrospinal Fluid Ubiquitin in Alzheimer's Disease and Parkinsonian Disorders

**DOI:** 10.1002/prca.201700100

**Published:** 2017-11-02

**Authors:** Simon Sjödin, Oskar Hansson, Annika Öhrfelt, Gunnar Brinkmalm, Henrik Zetterberg, Ann Brinkmalm, Kaj Blennow

**Affiliations:** ^1^ Department of Psychiatry and Neurochemistry Institute of Neuroscience and Physiology The Sahlgrenska Academy at University of Gothenburg Mölndal Sweden; ^2^ Clinical Memory Research Unit Department of Clinical Sciences Malmö Lund University Lund Sweden; ^3^ Memory Clinic Skåne University Hospital Malmö Sweden; ^4^ Clinical Neurochemistry Laboratory Sahlgrenska University Hospital Mölndal Sweden; ^5^ Department of Molecular Neuroscience University College London Institute of Neurology London UK; ^6^ UK Dementia Research Institute at UCL London UK

**Keywords:** alzheimer's disease, biomarker, parkinson's disease, progressive supranuclear palsy, ubiquitin

## Abstract

**Purpose:**

Dysfunctional proteostasis, with decreased protein degradation and an accumulation of ubiquitin into aggregated protein inclusions, is a feature of neurodegenerative diseases. Identifying new potential biomarkers in cerebrospinal fluid (CSF) reflecting this process could contribute important information on pathophysiology.

**Experimental design:**

A developed method combining SPE and PRM‐MS is employed to monitor the concentration of ubiquitin in CSF from subjects with Alzheimer's disease (AD), Parkinson's disease (PD), and progressive supranuclear palsy (PSP). Four independent cross‐sectional studies are conducted, studies 1–4, including controls (*n* = 86) and participants with AD (*n* = 60), PD (*n* = 15), and PSP (*n* = 11).

**Results:**

The method shows a repeatability and intermediate precision not exceeding 6.1 and 7.9%, respectively. The determined LOD is 0.1 nm and the LOQ range between 0.625 and 80 nm. The CSF ubiquitin concentration is 1.2–1.5‐fold higher in AD patients compared with controls in the three independent AD‐control studies (Study 1, *p* < 0.001; Study 2, *p* < 0.001; and Study 3, *p* = 0.003). In the fourth study, there is no difference in PD or PSP, compared to controls.

**Conclusion and clinical relevance:**

CSF ubiquitin may reflect dysfunctional proteostasis in AD. The described method can be used for further exploration of ubiquitin as a potential biomarker in neurodegenerative diseases.

## 1 Introduction

Neuropathological investigations in neurodegenerative diseases typically reveal accumulation of aggregated proteins or peptides. In Alzheimer's disease (AD) these inclusions are extracellular plaques and intraneuronal tangles composed of aggregated amyloid β peptide (Aβ)^1^ and hyperphosphorylated tau protein,^2^, ^3^, ^4^ respectively. Tangles containing tau are also found in progressive supranuclear palsy (PSP).^5^ The main constituent of Lewy bodies in Parkinson's disease (PD) is an aggregated form of α‐synuclein.^6^ Interestingly, all of these inclusions, plaques,^7^, ^8^ tangles,^7^, ^8^, ^9^ and Lewy bodies^7^, ^10^ are positive for the highly conserved polypeptide ubiquitin.

Ubiquitin is fundamental in ATP‐dependent degradation of proteins^11^ by labeling of target substrates for proteolysis by the 26S proteasome.^12^, ^13^ Ubiquitin is conjugated to protein substrates^14^, ^15^ through the action of E1 activating enzymes,^16^ E2 conjugating enzymes, and E3 ligases.^17^ These enzymes produce an isopeptide bond between the N‐terminal Gly of ubiquitin and, for example, the ϵ‐NH_2_ group of Lys in substrate proteins.^14^

Decreased proteolysis with aging^18^ and the inhibitory effect of protein aggregates,^19^ tau,^20^ and Aβ^21^, ^22^, ^23^ on the proteasome suggests that proteostasis may be impaired in neurodegenerative diseases. Indeed, proteolytic activity of the proteasome has been found to be reduced in the hippocampus and parahippocampal gyrus, superior and middle temporal gyri, inferior parietal lobule,^24^ and gyrus rectus^20^ in AD. The level of intracellular ubiquitin is regulated by a balance of expression and degradation of a pool of free ubiquitin, free polyubiquitin chains, and conjugated mono‐ and polyubiquitin.^25^ In AD, an increased cerebral cortical level of ubiquitin in grey^26^, ^27^ and white matter^27^ has been found. Furthermore, the concentration of ubiquitin has previously been shown to be increased in cerebrospinal fluid (CSF) from subjects with AD^28^, ^29^, ^30^ and Creutzfeldt–Jakob disease (CJD).^30^, ^31^, ^32^

**Clinical Relevance**

We have developed and validated a method combining SPE and PRM–MS for monitoring the concentration of monomeric ubiquitin in human CSF. Ubiquitin is important in maintaining proteostasis by targeting substrates for degradation by the proteasome. However, in neurodegenerative diseases protein aggregates interfere with proteasomal degradation. Also, within the brain, protein aggregates and inclusions, characteristic for these diseases are labeled by ubiquitin. Collectively, this may suggest ubiquitin to reflect disrupted proteolysis in neurodegenerative diseases. Here, we have identified significantly increased concentration of CSF ubiquitin in AD compared to controls. No difference was identified in PD or PSP compared to controls. Thus, CSF ubiquitin concentration may reflect dysfunctional proteostasis in AD. Using the developed method presented herein, ubiquitin as a potential biomarker can be explored in additional cohorts as well as in longitudinal studies to determine when, during the course of the disease, the concentration of CSF ubiquitin increases.

CSF offers a potent source for identification of biomarkers reflecting disease in the central nervous system. In AD, the CSF core biomarkers; the 42 amino acid long Aβ peptide (Aβ_1–42_), total tau protein (T‐tau), and phosphorylated tau protein (P‐tau),^33^ provides robust discrimination of patients with AD compared to controls as well as patients with mild cognitive impairment due to AD compared to stable mild cognitive impairment.^34^ Identifying additional CSF biomarkers would be of importance for the understanding of pathophysiological processes involved in neurodegenerative diseases. In turn, such biomarkers may improve detection of disease at an early stage; serve as tools to monitor disease progression or novel treatment strategies.

The development of hybrid high resolution mass spectrometers^35^, ^36^ has facilitated highly accurate^37^, ^38^ and selective^39^ measurements by PRM.^40^ In PRM, a precursor ion is fragmented and all product ions are monitored in parallel by MS/MS. Acquisition by MS/MS provides identification of the analyte with the quantitative benefits of post‐acquisition processing and optimization.^41^ Alternatively, SRM make use of triple quadrupole mass spectrometers, where the acquisition of a preselected transition occurs after sequential isolation of a precursor and fragment ion.^42^ SRM has proven useful in targeted proteomics^42^ and offers the potential of highly reproducible measurements.^43^ SRM and PRM have been shown to provide similar linearity,^41^, ^44^ dynamic range,^40^, ^41^, ^44^ LOQ,^41^, ^44^ and precision.^44^

A recent paper presented a novel SRM–MS method for quantification of ubiquitin in CSF, and reported increased ubiquitin CSF levels in AD and CJD.^30^ Here, we have applied PRM–MS and developed a method to accurately measure ubiquitin in CSF. The aim of the current investigation was to develop a method to measure CSF ubiquitin concentration and explore ubiquitin as potential biomarker in neurodegenerative disease. As our means of doing this, we developed an SPE micro‐HPLC PRM–MS method. The method was validated with excellent precision and a defined LOD/LOQ. Following analytical validation, the concentration of ubiquitin in CSF was measured in four independent cross‐sectional studies including controls (*n* = 86) and participants with AD (*n* = 60), PD (*n* = 15), and PSP (*n* = 11). An increased concentration of CSF ubiquitin was identified in AD but not in PD or PSP compared to controls, suggesting ubiquitin to be a measure of dysfunctional proteostasis in AD.

## 2 Experimental Section

### 2.1 Study Participants

CSF samples from four sets of cross‐sectional studies were used. Demographics of included subjects are presented in **Table** 1. Participants with AD in the studies had an AD CSF core biomarker profile according to the International Working Group 2 (IWG‐2) biomarker criteria^45^ with a low concentration of Aβ_1–42_ (<550 ng L^–1^) in combination with a high level of T‐tau (>400 ng L^–1^) or P‐tau phosphorylated at Thr181 (P‐tau_181_; >80ng L^–1^). Participants in study 1 (control, *n* = 15 and AD, *n* = 9) and 2 (control, *n* = 15 and AD, *n* = 14) were defined and selected solely by the IWG‐2 biomarker criteria.^45^ These samples were received when being analyzed in clinical routine setting at the Clinical Neurochemistry Laboratory, Mölndal, Sweden. Study 3 and 4 included a sub‐population from the Swedish BioFINDER study (www.biofinder.se). All individuals underwent examination by a study nurse and a medical doctor experienced in neurodegenerative disorders. Cognitive testing, psychiatric, and neurological assessments were performed, in addition to brain imaging and collection of CSF and blood. Study 3 included participants with AD dementia (*n* = 37), according to the National Institute of Neurological and Communicative Disorders and Stroke and the Alzheimer's Disease and Related Disorders Association^46^ and the IWG‐2 biomarker criteria.^45^ Cognitively healthy volunteers were included in the control group (*n* = 45). Study 4 consisted of participants diagnosed with PD (*n* = 15), according to the Neurological Disorders and Stroke diagnostic criteria for PD,^47^ PSP (*n* = 11), according to the National Institute of Neurological Disorders and Stroke–Society for Progressive Supranuclear Palsy International Workshop criteria.^48^ Healthy volunteers constituted the control group (*n* = 11). All controls underwent cognitive testing and neurologic examination by a medical doctor and individuals with objective cognitive or Parkinsonian symptoms were not included as controls in the present study. In studies 3 and 4 participants were recruited at Skåne University Hospital, Sweden, after providing written informed consent. Recruitment and inclusion of participants in this investigation were performed in accordance with regional ethical approvals given by the Regional Ethical Review Boards in Gothenburg and Lund, respectively.

**Table 1:** Demographics of cross‐sectional studies 1–4.

	Study 1a)		Study 2a)		Study 3b)		Study 4b)		
	Control	AD	Control	AD	Control	AD	Control	PD	PSP
**No. (M/F)**	15 (9/6)	9 (5/4)	15 (11/4)	14 (6/8)	45 (15/30)	37 (13/24)	11 (6/5)	15 (8/7)	11 (5/6)
**Age, median (IQR)**	71 (12)	75 (12)	74 (12)	75 (9)	75 (11)	73 (10)	69 (3)	69 (10)	71 (7)
**Ubiquitin, median (IQR), nm**	7.3 (2.8)	11 (3.3)c)	8.1 (4.2)	11 (3.6)c)	8.4 (3.0)	10 (3.7)d)	7.0 (2.6)	6.0 (2.0)	7.6 (2.6)
**T‐tau, median (IQR), ng L^–1^**	240 (92)	1050 (280)c)	310 (90)	890 (440)c)	320 (120)	700 (360)c)	250 (140)	220 (200)	290 (150)
**P‐tau_181_, median (IQR), ng L^–1^**	38 (11)	91 (10)c)	46 (24)	96 (32)c)	46 (15)	75 (35)c)	43 (17)	38 (15)	41 (34)
**Aβ_1‐42_, median (IQR), ng L^–1^**	980 (320)	360 (110)c)	800 (190)	370 (150)c)	910 (490)	370 (130)c)	1010 (230)	690 (420)e)	550 (450)f)

a) Includes subjects selected by the concentration of the AD core biomarkers Aβ_1‐42_, T‐tau, and P‐tau_181_; b) Includes subjects who have been clinically characterized and healthy volunteers as controls; c) Wilcoxon rank‐sum test, *p *< 0.001 versus control; d) Wilcoxon rank‐sum test, *p* < 0.01 versus control; e) Kruskal–Wallis test with Dunn post‐hoc, *p* < 0.05 versus control; f) Kruskal–Wallis test with Dunn post‐hoc, *p* < 0.01 versus control; AD, Alzheimer's disease; PD, Parkinson's disease; PSP, progressive supranuclear palsy; IQR, interquartile range.

### 2.2 CSF Samples

CSF was collected from participants by lumbar puncture in a standardized manner.^49^ CSF AD core biomarker concentrations; Aβ_1‐42_, T‐tau, and P‐tau_181_ were measured using commercially available enzyme‐linked immunosorbent assays (INNOTEST β‐AMYLOID(1‐42), INNOTEST hTAU Ag, and INNOTEST PHOSPHO‐TAU(181P); Fujirebio Europe, Ghent, Belgium). Additional information on quality control (QC) CSF pool samples is found in Supporting Information.

### 2.3 Protein Standards and Calibration Curve

Bovine ubiquitin (^12^C‐ubiquitin; average mass 8565 Da; 100% protein purity by SDS electrophoresis; Sigma–Aldrich Co., Saint Louis, MO, USA) and recombinant human ^13^C‐ and ^15^N‐ubiquitin (average mass 8940 and 8669 Da; >90% protein purity by SDS electrophoresis and >98% isotope enrichment purity; Silantes, GmbH, München, Germany) were dissolved in H_2_O to a concentration of 120 μm bovine ubiquitin, 100 μm ^13^C‐ubiquitin, and 100 μm ^15^N‐ubiquitin after correction for purity, 100 and 90%, respectively. The dissolved protein standards were aliquoted and stored at −80 °C. Protein standards were diluted in a dilution buffer containing 150 mm Na, 3 mm K, 1.4 mm Ca, 0.8 mm Mg, 0.7 mm P, 160 mm Cl, and 3.8 μm BSA (average mass 66430 Da; 100% purity by agarose electrophoresis; Sigma–Aldrich Co.). A 20 nm internal standard ^13^C‐ubiquitin solvent was prepared in dilution buffer for addition to CSF samples and QC pool samples. A protein standard dilution for the calibration curve was prepared by serially diluting ^15^N‐ubiquitin in the dilution buffer to 80, 40, 20, 10, 5, 2.5, 1.25, and 0.625 nm, respectively, as well as by adding ^13^C‐ubiquitin to a constant concentration of 20 nm at each calibration point. The calibration curves of the SPE PRM–MS method were evaluated as described by Almeida et al.^50^ Using linear regression, homoscedasticity was evaluated by *F*‐test and residual plots. Applying different weighting regimes, homoscedasticity and the sum percent relative error of calculated to nominal concentrations were monitored. LOD was calculated according to the guidelines provided by the international conference on harmonization by using the SD of the intercept and the slope.^51^ LOQ was defined as the concentrations at which the relative error of measured to nominal concentration did not exceed 15% and was calculated using the calibration curves.

### 2.4 Sample Preparation and SPE

For sample preparation, 50 μL 20 nm ^13^C‐ubiquitin internal standard was added to 50 μL CSF from samples and QC pools 1–3. SPE was performed using Oasis HLB 96‐well μElution Plates (2 mg sorbent and 30 μm particle size; Waters Co., Milford, MA, USA). Briefly, (1) the wells were conditioned by 2 × 300 μL methanol, (2) equilibrated with 2 × 300 μL H_2_O, (3) the samples were loaded, (4) washed by 2 × 300 μL H_2_O, (5) followed by 2 × 100 μL 30% methanol, and (6) finally eluted in 2 × 50 μL 70% methanol. The calibration curve was prepared using QC pool 1 by adding 50 μL from each of the eight calibration curve standard dilutions containing ^13^C‐ and ^15^N‐ubiquitin to 50 μL CSF. This was followed by SPE as described above. With each sample preparation and SPE plate, two technical replicate calibration curves were prepared as well as eight replicate samples each from at least two of the QC pools. Additional information on the evaluation of SPE by Western blotting is found in Supporting Information.

### 2.5 Quantification by PRM–MS

Forty microliters of the SPE eluted samples were injected and separated by HPLC on a Dionex UltiMate 3000 standard‐LC system (Thermo Fisher Scientific Inc.) and a Hypersil GOLD HPLC C18 column (length 100 mm; id 2.1 mm; particle size 1.9 μm; Thermo Fisher Scientific Inc.). Mobile phases were A: 0.1% formic acid in H_2_O (v/v) and B: 0.1% formic acid and 84% ACN in H_2_O (v/v). Separation was performed at a flow rate of 600 μL min^–1^ at +50 °C with a gradient going from 0 to 60% B over 3 min. The LC was operated in online mode coupled to a hybrid quadrupole‐OrbiTrap mass spectrometer (Q Exactive; Thermo Fisher Scientific Inc.) using ESI in positive ion mode. ESI was performed using a HESI‐II ionization probe (Thermo Fisher Scientific Inc.). By direct infusion of bovine ubiquitin at a flow rate of 600 μL min^–1^ the ionization settings were optimized as follows: a spray voltage of +4.1 kV; a heater temperature of +400 °C; a capillary transfer tube temperature of +380 °C; a sheath gas flow rate of 25; an auxiliary gas flow rate of 10; and an S‐lens RF level of 60. Ubiquitin was targeted using PRM by isolation of [M + 8H]^8+^ ubiquitin centered on *m/z* 1071.5 (^12^C‐ubiquitin), m/z 1084 (^15^N‐ubiquitin), and *m/z* 1117.5 (^13^C‐ubiquitin), respectively, using an isolation width of *m/z* 3.

Acquisition was performed by collecting single micro‐scans at a resolution setting of 35 000 at *m/z* 200, an automatic gain control target value of 1 × 10^6^, and a maximum injection time of 115 ms. The normalized collision energy (NCE) used for dissociation of precursor into product ions in the “higher‐energy collision” cell was optimized by direct infusion of ubiquitin while scanning the NCE as well as by LC–MS/MS by multiple injections of a ubiquitin solution where the NCE level was varied. The optimal NCE was determined as the energy resulting in the highest intensity of product ions normalized to the precursor ion.

### 2.6 Sample Analysis

For each set or plate of samples the analysis was initiated and ended with a calibration curve. Half the number of QC samples was analyzed prior to the set of subject samples and the remaining QC samples after the subject samples.

A processing method was developed using Thermo Xcalibur v2.2 (Thermo Fisher Scientific Inc.) which integrated the area of the product ions b_18_^2+^, y_24_^3+^, y_40_^4+^, y_43_^4+^, y_53_^5+^, y_58_^5+^, and y_59_^5+^. The mono‐isotopic *m/z* and the area integration *m/z* range for the targeted ions are presented in Supporting Information Table S1. The peak detection and integration was performed using the ICIS algorithm and the following settings; a retention time window of 10 s, 1 smoothing point, a baseline window of 40, an area noise factor of 5, a peak noise factor of 10, and detecting the highest peak with S/N of 3.

### 2.7 Statistics

Statistics were calculated and graphs created using GraphPad Prism v7.02 (GraphPad Software, Inc., La Jolla, CA, USA). Normal distribution was assessed by boxplots, histograms, and Shapiro–Wilk *W*‐test. For comparison of groups Kruskal–Wallis test with Dunn's test for multiple comparisons, comparing all pairs, or Wilcoxon rank‐sum test were used as indicated. A *p*‐value ≤ 0.05 was considered a significant result. ROC curves with calculated AUC were used to evaluate sensitivity and specificity. The AUC was considered significantly different from 0.5 when *p* ≤ 0.05. Correlations were calculated by determining Spearman's ρ where ρ ≥ 0.65 and a *p*‐value ≤ 0.01 indicate a correlation.

## 3 Results

### 3.1 Evaluation of the Calibration Curve

Fourteen calibration curves were evaluated as previously described.^50^ Applying weighting by 1/x^2^ achieved homoscedasticity and the least sum percent relative error of calculated to nominal concentrations. With each analysis, a pair of calibration curves was analyzed. The LOQ covered the full concentration range of calibration curves as the relative error of measured to nominal concentration did not exceed 15% for any of the pairs of calibration curves. Thus, the LOQ ranged from 0.625 to 80 nm. The pair of calibration curves generating the highest LOD, 0.1 nm, calculated using the SD of the intercept and the slope,^51^ with *r* ^2^ = 0.99 after weighting by 1/x^2^, was defined by the following linear equation:

(1) y=−0.007395% confidence  interval  CI ±0.0051×x+0.03595% CI ±0.0034.

The calibration curve is shown in Supporting Information Figure S1.

### 3.2 Method Development and Assay Precision

Ubiquitin was found to adhere and elute from the SPE HLB sorbent as visualized by Western blotting. Ubiquitin was eluted by methanol ranging in concentration from 30–80%; see Supporting Information Figure S2.

A PRM method was developed which isolate the [M + 8H]^8+^ precursor ions of endogenous ubiquitin (^12^C‐ubiquitin), ^13^C‐, and ^15^N‐ubiquitin. Precursor mass spectrum and MS/MS spectra of ubiquitin are shown in Supporting Information Figure S3 and S4, respectively. Extracted ion chromatograms of the targeted product ions showed co‐elution of ^12^C‐ and ^13^C‐ubiquitin, and ^15^N‐ and ^13^C‐ubiquitin, see Supporting Information Figure S5. Furthermore, as seen in the MS/MS spectra shown in Supporting Information Figures S6–8, there were no interferences in the *m/z* ranges for integrating peak areas of targeted product ions. The number of acquisitions across a chromatographic peak was 11.

Methodological precision was evaluated by calculating repeatability and intermediate precision. QC pool 1 and 2 were analyzed on seven separate occasions while pool 3 was analyzed on five occasions. On each occasion a minimum of eight technical replicates were prepared. Using ANOVA, the repeatability was found to be 2.8, 3.6, and 6.1% and the intermediate precision 7.0, 6.5, and 7.9%, for QC pool 1, 2, and 3, respectively.

### 3.3 CSF Ubiquitin Concentration in Alzheimer's Disease Core Biomarker Profile Samples

**Figure 1:**
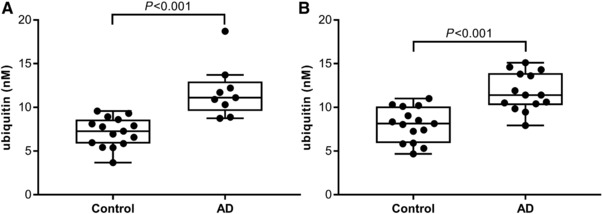
Ubiquitin concentrations in AD core CSF biomarker profile samples. Two cross‐sectional studies were performed including subjects selected by their AD core CSF biomarker profile. A) Study 1 included subjects designated as controls (*n* = 15) and AD (*n* = 9). B) Study 2 included subjects designated as controls (*n* = 15) and AD (*n* = 14). The groups were compared using Wilcoxon rank‐sum test.

In Study 1, a significantly higher concentration of ubiquitin was found in subjects with AD compared to controls (*p *< 0.001), see **Figure** 1A. In Study 1, two technical replicates were analyzed from each sample on a single occasion. The average concentration of the replicates was used for group comparison. The CV for all of the technical replicates analyzed did not exceed 10%; see Supporting Information Figure S9. A correlation between T‐tau and ubiquitin (ρ = 0.69, *p* = 0.006; Supporting Information Figure S10A) and P‐tau_181_ and ubiquitin (ρ = 0.75, *p* = 0.002; Supporting Information Figure S10B) was found in the control group. However, no correlation was found between Aβ_1–42_ and ubiquitin; see Supporting Information Figure S10C. There were no correlations between Aβ_1–42_, T‐tau, or P‐tau_181_ and ubiquitin in the AD group; see Supporting Information Figure S11.

In Study 2, a significant higher concentration of ubiquitin was identified in the AD group compared to the control group (*p* < 0.001), see Figure 1B. In the control group correlations were found between T‐tau and ubiquitin (ρ = 0.84, *p* < 0.001) and P‐tau_181_ and ubiquitin (ρ = 0.80, *p *< 0.001), but not between Aβ_1–42_ and ubiquitin; see Supporting Information Figure S12. In the control group, a correlation was also identified between T‐tau and P‐tau_181_ (ρ = 0.84, *p* < 0.001), data not shown. In the AD group, there were no correlations identified between Aβ_1‐42_, T‐tau, or P‐tau_181_ and ubiquitin; see Supporting Information Figure S13. However, there was a correlation between T‐tau and P‐tau_181_ (ρ = 0.88, *p* < 0.001), data not shown.

### 3.4 CSF Ubiquitin Concentration in Clinically Characterized Participants with AD, PD, and PSP

**Figure 2:**
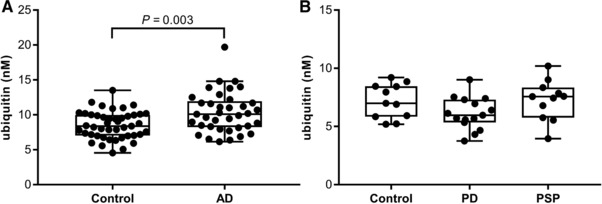
CSF ubiquitin concentrations in participants with AD, PD, and PSP. Two cross‐sectional studies were performed including clinically characterized participants. The participants with AD met the AD core CSF biomarker criteria suggesting AD. A) Study 3 included healthy volunteers (controls, *n* = 45) and participants with AD (*n* = 37). The groups were compared using Wilcoxon rank‐sum test. B) Study 4 included a control group (*n* = 11), participants with PD (*n* = 15) and PSP (*n* = 11). The groups were compared using Kruskal–Wallis with Dunn's multiple comparison test, comparing all pairs.

**Figure 3:**
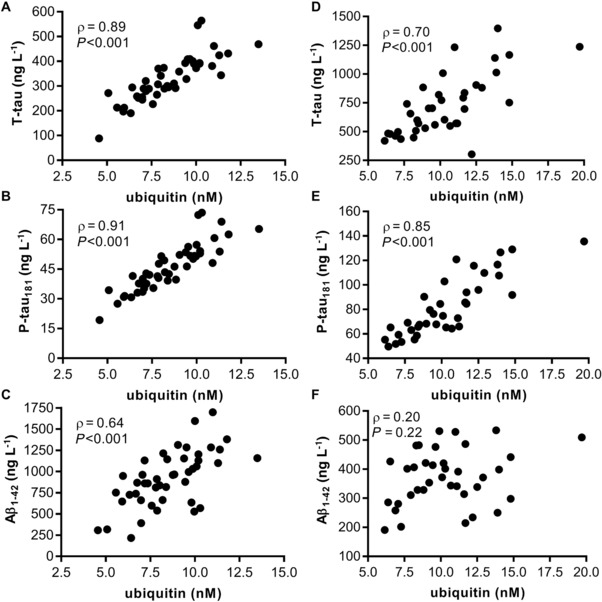
Correlations of CSF ubiquitin with AD core CSF biomarker concentrations. Correlations were calculated between the concentration of ubiquitin and the AD core CSF biomarkers; T‐tau, P‐tau_181_, or the 42 amino acid long Aβ_1–42_. Indicated are Spearman's ρ and the *p*‐value. The scatter plots show the concentrations in controls (*n* = 45), A–C, and in participants with AD (*n* = 37), D–F, from Study 3.

In Study 3, a significantly higher concentration of ubiquitin was found in the AD group compared to the control group (*p* = 0.003), see **Figure** 2A. In the control group, correlations were found between T‐tau and ubiquitin (ρ = 0.89, *p* < 0.001) and P‐tau_181_ and ubiquitin (ρ = 0.91, *p* < 0.001), see **Figure** 3A and B, respectively. For Aβ_1–42_ and ubiquitin in the control group ρ = 0.64 with *p* < 0.001, see Figure 3C. In the AD group, correlations were identified between T‐tau and ubiquitin (ρ = 0.70, *p* < 0.001) and P‐tau_181_ and ubiquitin (ρ = 0.85, *p* < 0.001), but not between Aβ_1–42_ and ubiquitin, see Figure 3D–F. In the control and AD group. there was also a correlation between T‐tau and P‐tau_181_, ρ = 0.93, *p* < 0.001 and ρ = 0.83, *p* < 0.001, respectively, data not shown. When comparing the AD group against the control group, the AUC for ubiquitin was found to be 0.69 (95% CI 0.57–0.81; *p* = 0.003) with a sensitivity and specificity of 62% (95% CI 45–76%) and 60% (95% CI 44–74%) at 9.1 nm ubiquitin, respectively; see Supporting Information Figure S14.

In Study 4, there were no significant differences in the concentration of ubiquitin between the control, PD, and PSP groups, see Figure 2B. In the PD group, correlations were identified between T‐tau and ubiquitin (ρ = 0.80, *p* < 0.001) and P‐tau_181_ and ubiquitin (ρ = 0.93, *p* < 0.001), but not between Aβ_1‐42_ and ubiquitin; see Supporting Information Figure S15. In the group of subjects with PSP, a correlation was found between P‐tau_181_ and ubiquitin (ρ = 0.82, *p* = 0.003) but not between T‐tau or Aβ_1‐42_ and ubiquitin; see Supporting Information Figure S16. In the PSP group, there were also correlations between T‐tau or P‐tau_181_ and age, ρ = 0.73, *p* = 0.01 and ρ = 0.73, *p* = 0.01, respectively, data not shown. In the control group, correlations were identified between T‐tau and ubiquitin (ρ = 0.87, *p* < 0.001) and P‐tau_181_ and ubiquitin (ρ = 0.84, *p* = 0.002) but not between Aβ_1‐42_ and ubiquitin; see Supporting Information Figure S17. For all three groups, PD, PSP, and controls, there was a correlation between T‐tau and P‐tau_181_ (ρ = 0.83, *p* < 0.001; ρ = 0.74, *p* = 0.01; and ρ = 0.98, *p* < 0.001, respectively), data not shown.

## 4 Discussion

In the present study, we evaluated CSF ubiquitin as a biomarker for neurodegenerative disorders. A method that selectively measures ubiquitin concentration was developed by combining SPE and PRM–MS. The method showed excellent precision and the LOQ/LOD was determined. Using the method, we identified a significantly increased concentration of ubiquitin in CSF from participants with AD compared to controls.

It has been shown that proteolysis decreases with aging^18^ and that proteins, peptides, and protein aggregates have the ability to inhibit the proteasome.^19^, ^20^, ^21^, ^22^, ^23^ A resulting disturbance of ubiquitin homeostasis might lead to an accumulation of ubiquitin within the brain.^26^, ^27^ The amount of ubiquitin has previously been shown to be elevated in CSF in AD.^28^, ^29^, ^30^ In PSP, the concentration of ubiquitin in CSF has been indicated to exist at both unaltered^30^ or increased concentrations^52^ compared to controls. We identified no significantly altered concentration of ubiquitin in PSP relative controls and PD. Neither did we identify significantly altered concentrations of ubiquitin in CSF from participants with PD compared to controls, which is in agreement with previous findings.^30^, ^52^ However, the concentration of ubiquitin was seemingly lower in participants with PD compared to controls and participants with PSP.

Oeckl et al.^30^ targeted CSF ubiquitin by adapting sample preparation by precipitation followed by SRM–MS. They defined a quantitative range between 0.234 and 23 nm with an intra‐ and interassay precision not exceeding 7.4 and 10.3%, respectively.^30^ We have employed SPE and high resolution PRM–MS and demonstrated a LOQ of 0.625–80 nm and reproducibility and intermediate precision not exceeding 6.1 and 7.9%, respectively. PRM^40^ offers highly accurate^37^, ^38^ and selective^39^ measurements and can successfully be applied to the analysis of complex samples.^40^, ^53^ With good precision, we have previously measured the lysosomal membrane protein LAMP2 in CSF using PRM.^54^ Furthermore, measurements using PRM have shown similar performance compared to SRM,^40^, ^41^, ^44^ however, with the advantage of a broader dynamic range.^40^

CSF ubiquitin concentration was higher in AD patients than in controls. The AD core CSF biomarkers discriminate AD from controls with high sensitivity and specificity, as previously shown.^55^ However, herein, a direct comparison between ubiquitin and the AD core biomarkers could not be performed as the AD core biomarkers were part of the inclusion criteria of the study. Furthermore, the sizes of the study populations were quite small limiting the ability to draw conclusions about sensitivity and specificity.

Similarly, correlations calculated in studies 1, 2, and 4 might be affected by the low number of participants. However, correlations were identified between the concentrations of ubiquitin and T‐tau or P‐tau_181_ in the groups of clinically characterized AD and controls in study 3 as well as in the control groups of studies 1 and 2. Interestingly, we also identified correlations between the concentration of ubiquitin and T‐tau or P‐tau_181_ in PD and the control group in study 4 as well as for ubiquitin and P‐tau_181_ in the PSP group. Previous investigations have revealed unaltered CSF concentrations of T‐tau and P‐tau in PD and PSP^56^ as well as lowered concentrations of T‐tau and P‐tau in PSP,^57^ compared to controls. Furthermore, brainstem tangles formed in PSP have shown a low degree of ubiquitin labeling.^58^, ^59^

Increased CSF ubiquitin concentration in AD might reflect increased level of ubiquitin in the brain,^26^, ^27^ as a result of dysfunctional proteostasis. The CSF concentration of ubiquitin has been shown to be significantly increased in CJD,^30^, ^31^, ^32^ another disease characterized by high CSF T‐tau concentration.^60^ The CSF concentrations of T‐tau is considered to reflect neuronal and axonal degeneration,^61^ while P‐tau_181_ has been found to correlate with tangle load in the brain.^62^ T‐tau has been suggested to reflect the intensity of neurodegeneration and to predict progression rate.^63^, ^64^, ^65^ We identified a strong correlation between CSF ubiquitin and T‐tau, which supports ubiquitin to also reflect neuronal and axonal degeneration. However, ubiquitin is a constituent of intra‐ and extracellular neuropathological inclusions such as plaques and tangles^7^, ^8^, ^9^ and might thus indicate such alterations. Indeed we identified a strong correlation between CSF ubiquitin and P‐tau. Ubiquitin has specifically been identified as a posttranslational modification of tau (UniProtKB:P10636‐8) amino acid residues Lys254,^66^, ^67^ Lys257,^66^ Lys311,^66^, ^67^ Lys317,^66^ and Lys353.^67^ The CSF concentration of Aβ_1–42_ is inversely correlated with plaque load.^68^ Although also being identified as a molecular component of plaques,^7^, ^8^ no correlation was found between the concentration of ubiquitin and Aβ_1–42_ in any of our four investigations.

Previous studies have shown a direct involvement of ubiquitin in neurodegenerative disease,^69^ exemplified by an ubiquitin C‐terminal extension^70^ shown to inhibit the ubiquitin‐proteasome system.^71^ Furthermore, the functionally diverse protein, UCH‐L1, a deubiquitinase, ubiquitin ligase, and stabilizer of ubiquitin,^72^ is a constituent of plaques and has been shown to exist a decreased levels in cortical brain tissue from subjects with AD and PD.^73^ UCH‐L1 also confers a genetic component in PD^74^, ^75^ and has been found to exist at decreased CSF concentration in PD, multiple system atrophy and PSP,^76^ and increased concentration in AD,^49^ compared to controls.

Identifying proteins or peptides as new potential biomarkers, reflecting biological systems with a pathological involvement in diseases of the central nervous system, is important for increasing our understanding of neurodegenerative diseases. Ubiquitin is fundamentally important in protein homeostasis and has been shown to be associated with neuropathological hallmarks in several neurodegenerative diseases. The ubiquitin‐proteasome system and proteostasis is compromised in aging and in neurodegenerative diseases which might be reflected by the concentration of ubiquitin in CSF. Using a developed method combining SPE and PRM–MS, we have identified increased CSF concentration of ubiquitin in AD compared to controls, whereas no alteration was found in PD and PSP. Measuring CSF ubiquitin concentration in additional cohorts and longitudinal studies using tools such as SPE PRM–MS is needed to further investigate and validate ubiquitin as a potential biomarker for AD.

**Abbreviations**
Aβ: amyloid β peptide
Aβ_1–42_: 42 amino acid long Aβ peptide
AD: Alzheimer's disease
CI: confidence interval
CJD: Creutzfeldt‐Jakob disease
CSF: cerebrospinal fluid
IWG‐2: International Working Group 2
NCE: normalized collision energy
PD: Parkinson's disease
P‐tau: phosphorylated tau protein
P‐tau_181_: tau protein phosphorylated at Thr181
PSP: progressive supranuclear palsy
QC: quality control
T‐tau: total tau protein

## Conflict of Interest

HZ and KB are co‐founders of Brain Biomarker Solutions in Gothenburg AB, a GU Ventures‐based platform company at the University of Gothenburg. HZ has served at advisory boards of Roche Diagnostics, Eli Lilly, and Pharmasum Therapeutics. KB has served as a consultant or at advisory boards for Alzheon, Eli Lilly, Fujirebio Europe, IBL International, and Roche Diagnostics. OH has acquired research support (for the institution) from Roche, GE Healthcare, Biogen, AVID Radiopharmaceuticals, Fujirebio, and Euroimmun. In the past 2 years, OH has received consultancy/speaker fees (paid to the institution) from Lilly, Roche, and Fujirebio. The other authors report no conflict of interest.

## Supporting information

Supporting Information

Supporting Figures

Table S1. Product Ions Used for Quantification.
