# Supplementary material for: Mass Spectrometric Analysis of Cerebrospinal Fluid Ubiquitin in Alzheimer's Disease and Parkinsonian Disorders
Source: Proteomics Clin Appl. 2017 Nov 2;11(11-12):1700100. doi: 10.1002/prca.201700100 (PMC5765402; doi:10.1002/prca.201700100)
Supplement: Supplementary file 1 — Supporting Information [file PRCA-11-na-s001.pdf]

Supporting Information

Mass Spectrometric Analysis of Cerebrospinal Fluid Ubiquitin in Alzheimer's Disease and Parkinsonian Disorders

Simon Sjödin[1], Oskar Hansson[2,3], Annika Öhrfelt[1], Gunnar Brinkmalm[1], Henrik Zetterberg[1,4,5,6], Ann Brinkmalm[1,4], Kaj Blennow[1,4]

[1]Department of Psychiatry and Neurochemistry, Institute of Neuroscience and Physiology, The Sahlgrenska Academy at University of Gothenburg, Mölndal, Sweden

[2]Clinical Memory Research Unit, Department of Clinical Sciences Malmö, Lund University, Lund, Sweden

[3]Memory Clinic, Skåne University Hospital, Malmö, Sweden

[4]Clinical Neurochemistry Laboratory, Sahlgrenska University Hospital, Mölndal, Sweden

[5]Department of Molecular Neuroscience, University College London Institute of Neurology, Queen Square, London, UK

[6]UK Dementia Research Institute at UCL, London, United Kingdom

Corresponding Author: Simon Sjödin, Department of Psychiatry and Neurochemistry, Institute of Neuroscience and Physiology, The Sahlgrenska Academy at University of Gothenburg, House V3, SU/Mölndal, SE-43180, Mölndal, Sweden. simon.sjodin@neuro.gu.se.

1 Materials and Methods

1.1 Quality Control CSF Pools

Three quality control (QC) cerebrospinal fluid (CSF) pools were prepared to monitor methodological performance; QC pool 1 (550ng/L of the 42 amino acid long Aβ peptide ($A\beta_{1-42}$), 410ng/L total tau protein (T-tau), 46ng/L tau protein phosphorylated at Thr181 ($P\text{-}tau_{181}$) and 7.9nM ubiquitin), QC pool 2 (540ng/L $A\beta_{1-42}$, 400ng/L T-tau, 46ng/L $P\text{-}tau_{181}$ and 7.7nM ubiquitin) and QC pool 3 (480ng/L $A\beta_{1-42}$, 470ng/L T-tau, 46ng/L $P\text{-}tau_{181}$ and 26nM ubiquitin) by pooling CSF samples. To QC pool 3, bovine ubiquitin from erythrocytes (^{12}C-ubiquitin; average mass 8565Da; 100% protein purity by SDS electrophoresis; Sigma-Aldrich Co., Saint Louis, MO, USA) was added to a final concentration of 10nM excluding existing endogenous ubiquitin. The QC CSF pools were aliquoted and stored at -80°C.

1.2 Evaluation of SPE by Western Blotting

CSF or 50mM NH_4HCO_3 was mixed 1:1 with 1µM ^{12}C-ubiquitin in H_2O. SPE was performed by using Oasis HLB 96-well µElution Plates (Waters Co.). The wells were conditioned by 2×300µL methanol, equilibrated with 2×300µL H_2O before the samples were loaded. Collection was made of samples not adhering to the HLB sorbent. Collection was then made of samples being eluted by a wash of 2×300µL H_2O. After washing the wells, samples were eluted in parallel with 2×100µL 10-100% methanol. The samples were dried by vacuum centrifugation. Western blotting was performed. Briefly, samples were dissolved in 1×NuPAGE LDS sample buffer (Thermo Fisher Scientific Inc., Waltham, MA, USA) with 50mM dithiothreitol and denatured by incubation at +95°C for 5min. The samples were separated on a Novex 4–12% tris–glycine gel (Thermo Fisher Scientific Inc.) and transferred to an Immobilion-P PVDF membrane (Merck KGaA, Darmstadt, Germany). The membrane was blocked in 5% non-fat milk in PBS with 0.05% Tween (PBS-T). Following blocking, the membrane was incubated with an anti-ubiquitin horseradish-peroxidase conjugated antibody (Ub Antibody P4D1; Santa Cruz Biotechnology, Inc., Dallas, TX, USA), diluted 1:5000 in 5% non-fat milk PBS-T for 1h at room temperature. The membrane was washed with PBS-T followed by incubation with ECL select (Amersham, GE Healthcare UK Ltd. Little Chalfont, UK) prior to detection of chemiluminescence using a Fujifilm LAS-3000 camera (Fuji Photo Film Co., Ltd., Tokyo, Japan) and the Image Reader LAS-3000 v2.2 software (Fuji Photo Film Co., Ltd.). The images were processed by Multi Gauge v3.0 (Fuji Photo Film Co., Ltd.).
