# Supplementary material for: Mass Spectrometric Analysis of Cerebrospinal Fluid Ubiquitin in Alzheimer's Disease and Parkinsonian Disorders
Source: Proteomics Clin Appl. 2017 Nov 2;11(11-12):1700100. doi: 10.1002/prca.201700100 (PMC5765402; doi:10.1002/prca.201700100)
Supplement: Supplementary file 2 — Supporting Figures [file PRCA-11-na-s002.pdf]

Supporting Information

Mass Spectrometric Analysis of Cerebrospinal Fluid Ubiquitin in Alzheimer's Disease and Parkinsonian Disorders

Simon Sjödin[1], Oskar Hansson[2,3], Annika Öhrfelt[1], Gunnar Brinkmalm[1], Henrik Zetterberg[1,4,5,6], Ann Brinkmalm[1,4], Kaj Blennow[1,4]

[1]Department of Psychiatry and Neurochemistry, Institute of Neuroscience and Physiology, The Sahlgrenska Academy at University of Gothenburg, Mölndal, Sweden

[2]Clinical Memory Research Unit, Department of Clinical Sciences Malmö, Lund University, Lund, Sweden

[3]Memory Clinic, Skåne University Hospital, Malmö, Sweden

[4]Clinical Neurochemistry Laboratory, Sahlgrenska University Hospital, Mölndal, Sweden

[5]Department of Molecular Neuroscience, University College London Institute of Neurology, Queen Square, London, UK

[6]UK Dementia Research Institute at UCL, London, United Kingdom

Corresponding Author: Simon Sjödin, Department of Psychiatry and Neurochemistry, Institute of Neuroscience and Physiology, The Sahlgrenska Academy at University of Gothenburg, House V3, SU/Mölndal, SE-43180, Mölndal, Sweden. simon.sjodin@neuro.gu.se.

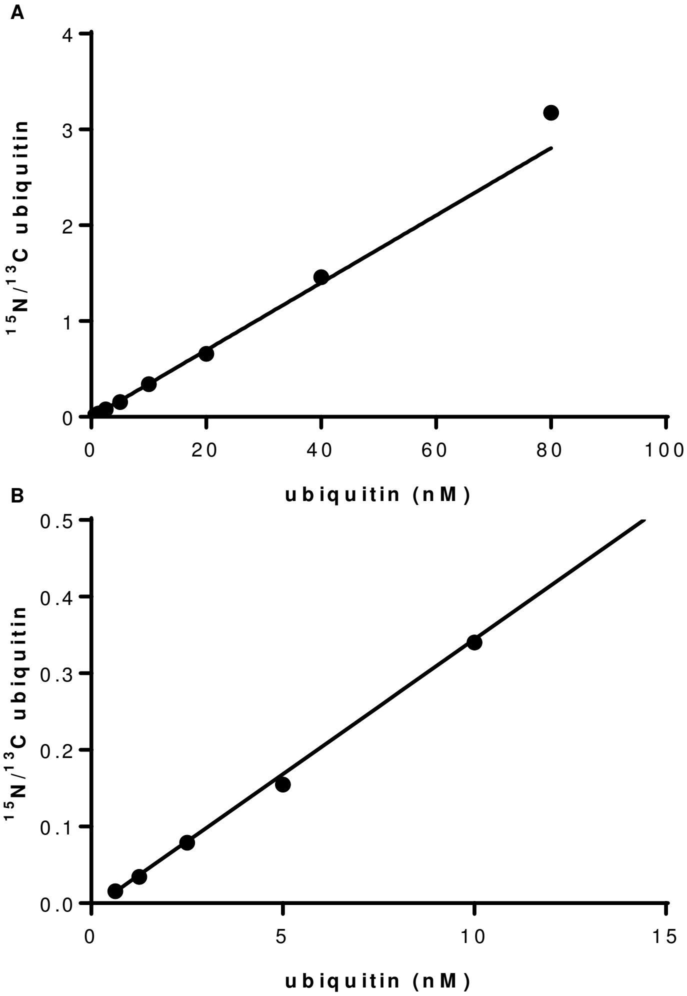

Figure S1. Calibration Curve for Ubiquitin in Cerebrospinal Fluid. A, Full range of the calibration curve. B, Magnification of the lower concentration range shown in a. Using weighting by $1/x^2$ a line was fitted by least-squares linear regression. The markers show the average of two technical replicate curves analyzed on a single occasion.

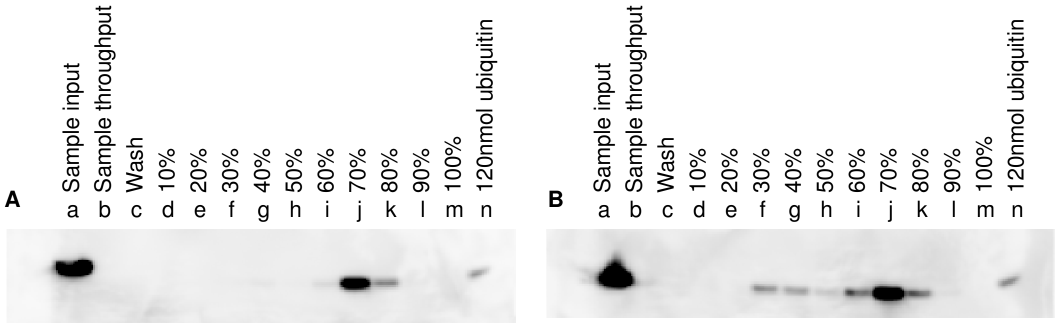

Figure S2. Preparation of Ubiquitin from Cerebrospinal Fluid Using SPE. Solid-phase extraction was performed to purify mono-ubiquitin from a, 50mM NH_4HCO_3 and b, cerebrospinal fluid. Bovine ubiquitin was added to both NH_4HCO_3 and cerebrospinal fluid prior to purification. The Western blots show sample input (A), sample not adhering to the HLB sorbent (B), sample washed through by 2×300μL H_2O (C), sample eluted by 10-100% methanol (D-M) and 120nmol bovine ubiquitin (N).

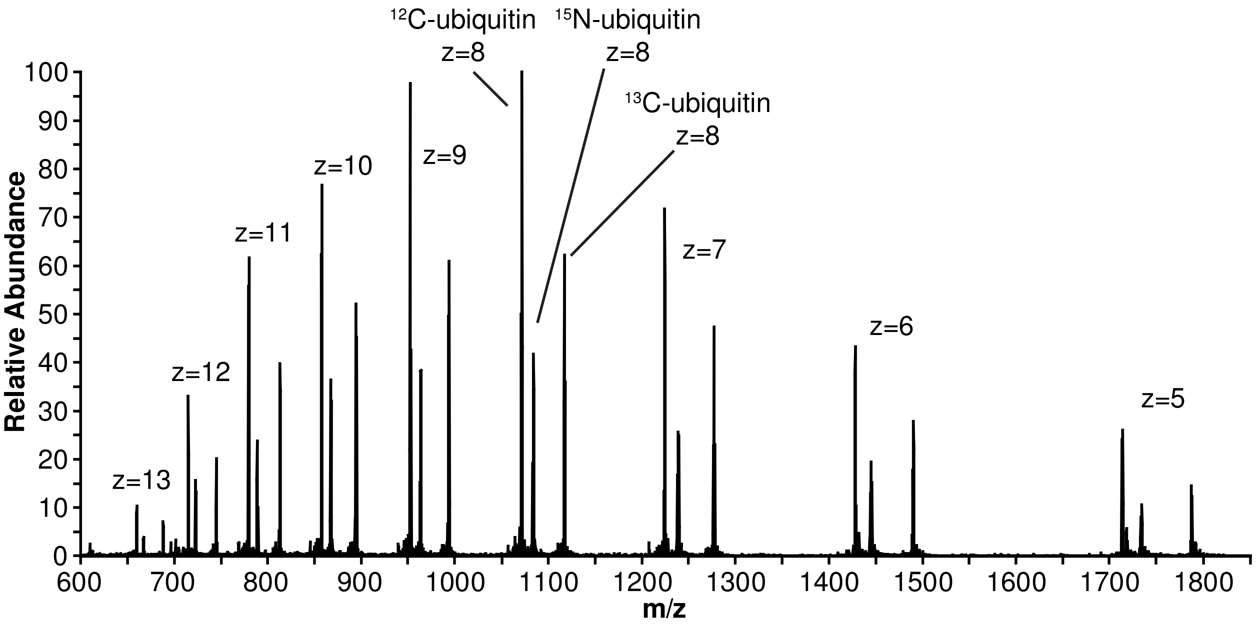

Figure S3. Precursor Mass Spectrum of Ubiquitin. Shown is a mixture of ^{12}C-ubiquitin, ^{13}C-ubiquitin and ^{15}N-ubiquitin with the charge state distribution clearly visible. z=charge state.

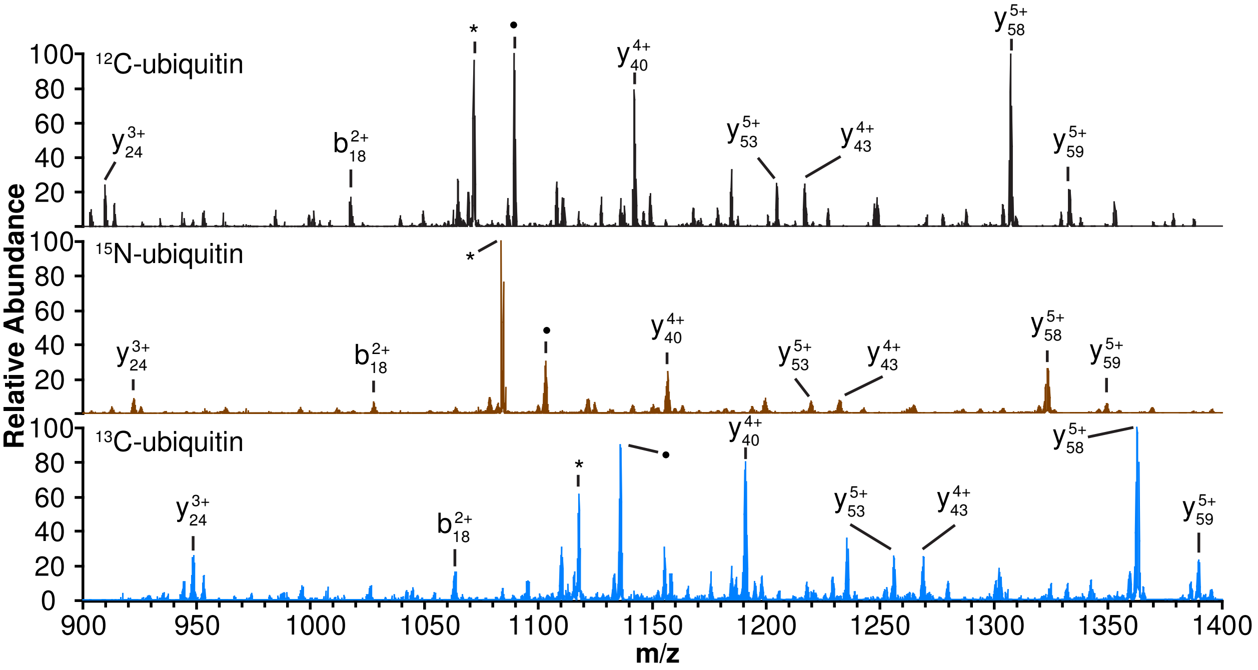

Figure S4. MS/MS of Ubiquitin. Indicated are the product ions from ^{12}C-, ^{13}C- and ^{15}N-ubiquitin used for quantification; b_{18}^{2+}, y_{24}^{3+}, y_{40}^{4+}, y_{43}^{4+}, y_{53}^{5+}, y_{58}^{5+} and y_{59}^{5+}. Also indicated are the precursor ions $[M+8H]^{8+}$ (*) and the y_{58}^{6+} product ions (•).

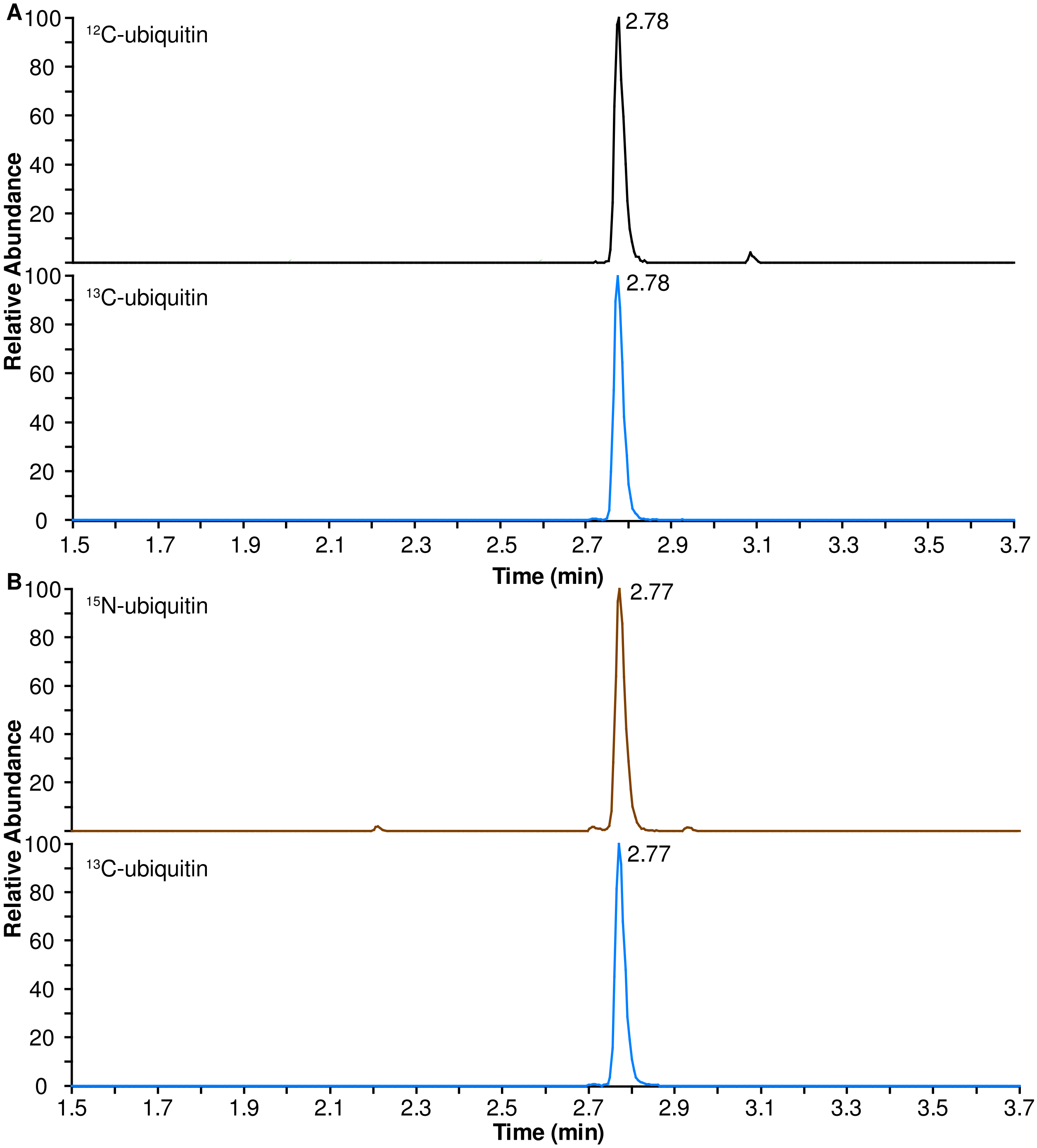

Figure S5. Extracted Ion Chromatogram for Targeted Product Ions. Shown are the combined signals of the targeted product ions b_{18}^{2+}, y_{24}^{3+}, y_{40}^{4+}, y_{43}^{4+}, y_{53}^{5+}, y_{58}^{5+} and y_{59}^{5+} for A, ^{12}C- and ^{13}C-ubiquitin, and B, ^{15}N- and ^{13}C-ubiquitin, respectively. Indicated are the retention times (min).

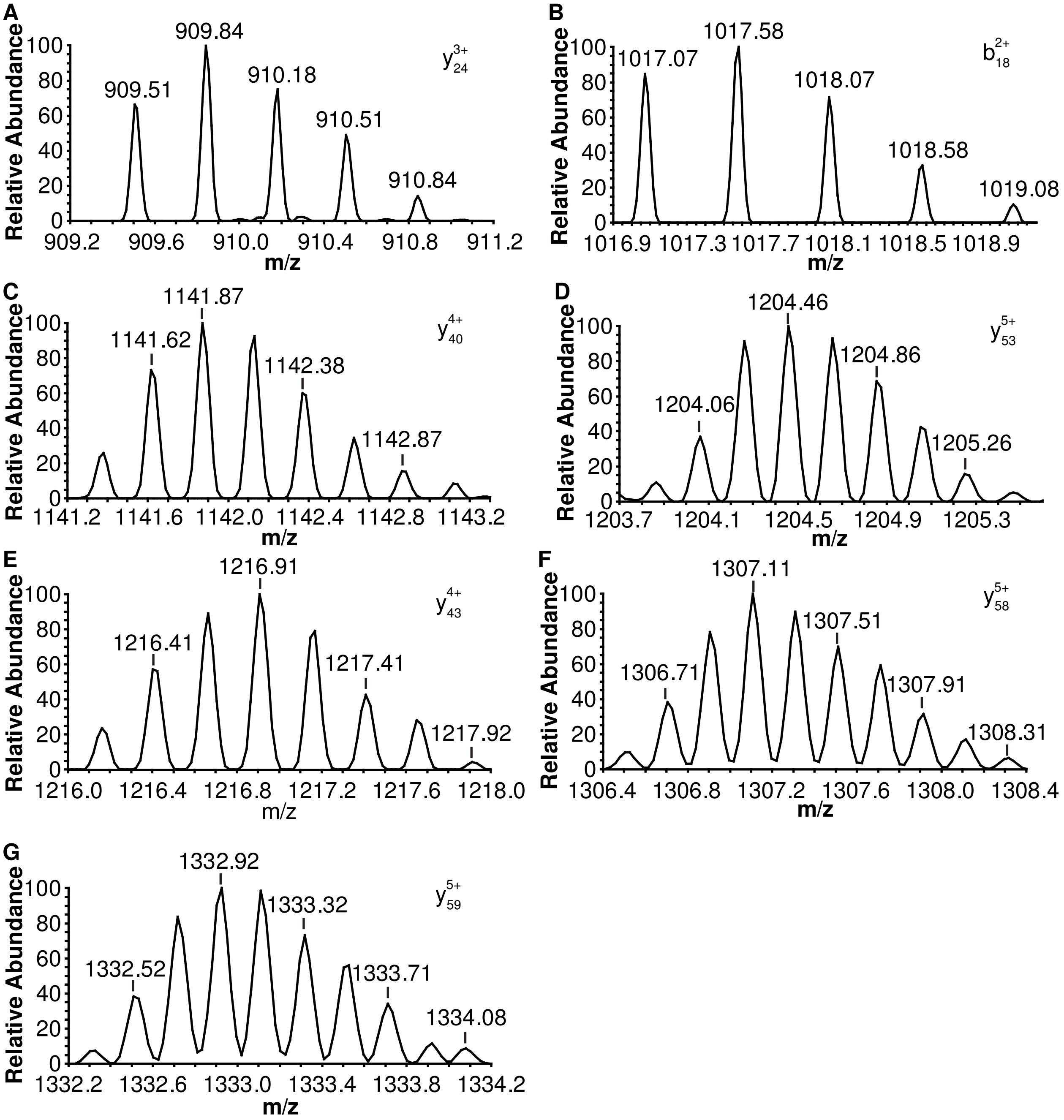

Figure S6. MS/MS of ^{12}C-Ubiquitin Product Ions. Shown are the product ions used for quantification; A, y_{24}^{3+}; B, b_{18}^{2+}; C, y_{40}^{4+}; D, y_{53}^{5+}; E, y_{43}^{4+}; F, y_{58}^{5+}; and G, y_{59}^{5+} in the *m/z* range used for peak area integration.

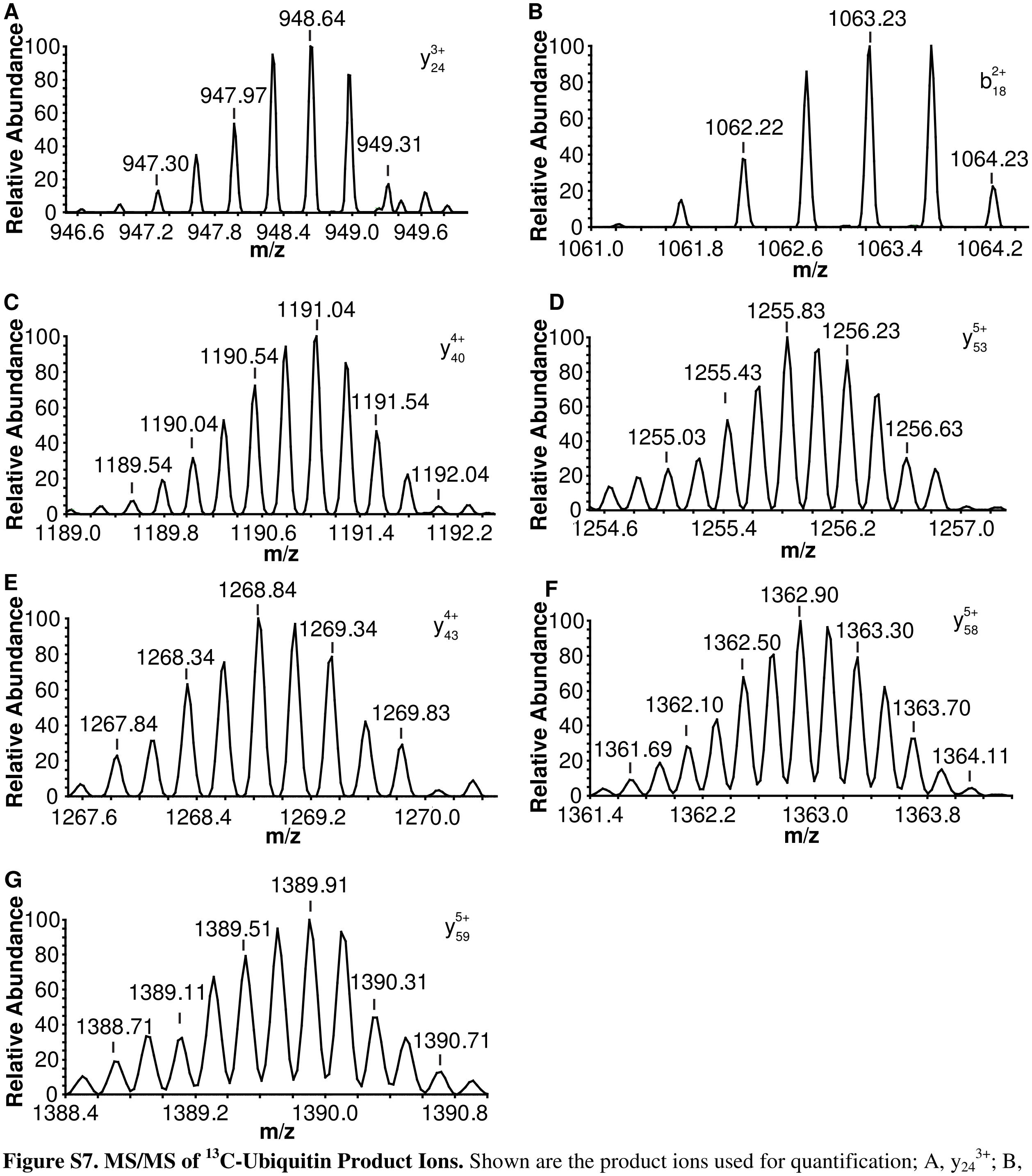

Figure S7. MS/MS of ^{13}C-Ubiquitin Product Ions. Shown are the product ions used for quantification; A, y_{24}^{3+}; B, b_{18}^{2+}; C, y_{40}^{4+}; D, y_{53}^{5+}; E, y_{43}^{4+}; F, y_{58}^{5+}; and G, y_{59}^{5+} in the *m/z* range used for peak area integration.

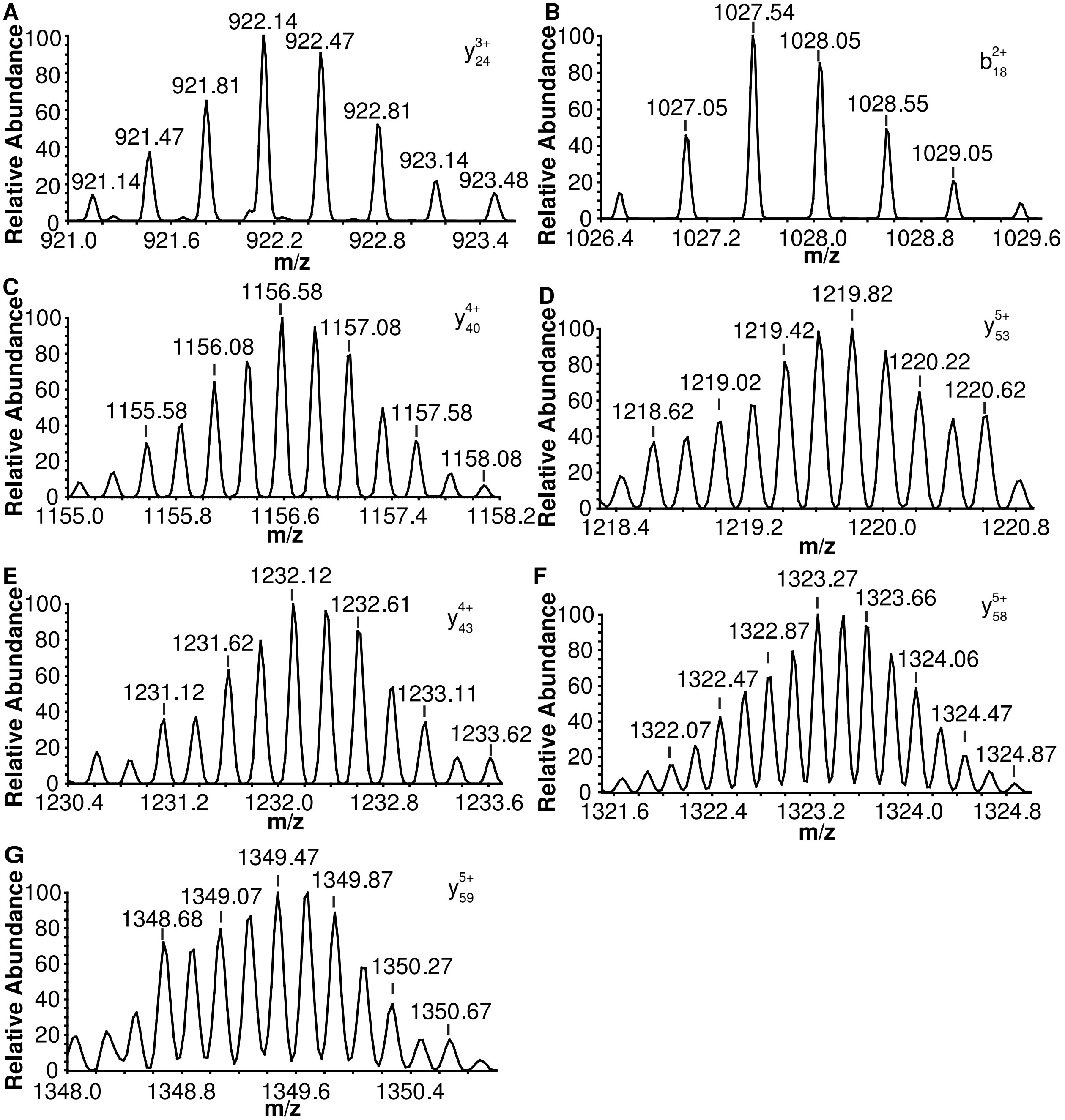

Figure S8. MS/MS of ^{15}N-Ubiquitin Product Ions. Shown are the product ions used for quantification; A, y_{24}^{3+}; B, b_{18}^{2+}; C, y_{40}^{4+}; D, y_{53}^{5+}; E, y_{43}^{4+}; F, y_{58}^{5+}; and G, y_{59}^{5+} in the *m/z* range used for peak area integration.

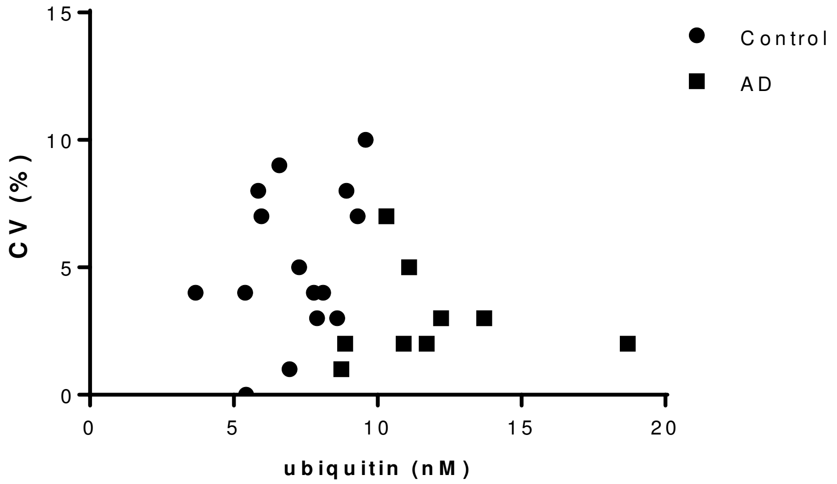

Figure S9. Repeatability of Ubiquitin Concentration Measurements in Study 1. Shown are the coefficient of variation (CV) and the average concentration of two replicates analyzed for each subject included in Study 1. Alzheimer's disease (AD; n=9) and controls (n=15).

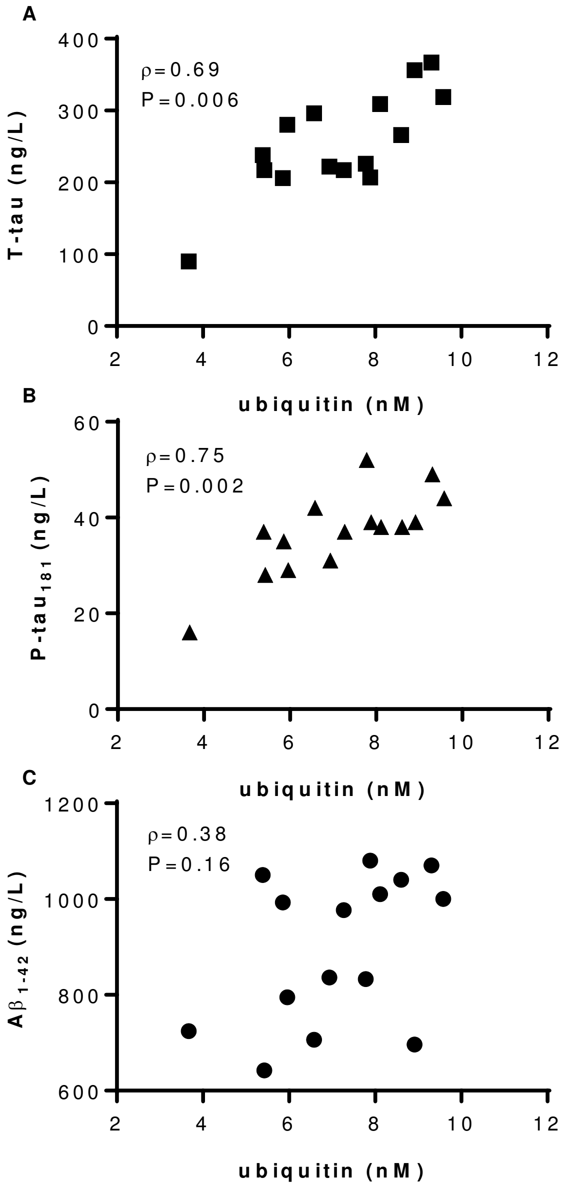

Figure S10. Correlations of CSF Ubiquitin with CSF Core Biomarkers in Controls in Study 1. Shown are scatter plots for the concentration of ubiquitin and A, total tau protein (T-tau); B, tau protein phosphorylated at Thr181 (P-tau_{181}) or; C, the 42 amino acid long amyloid β peptide ($A\beta_{1-42}$). Indicated are Spearman's ρ and the P-value. n=15.

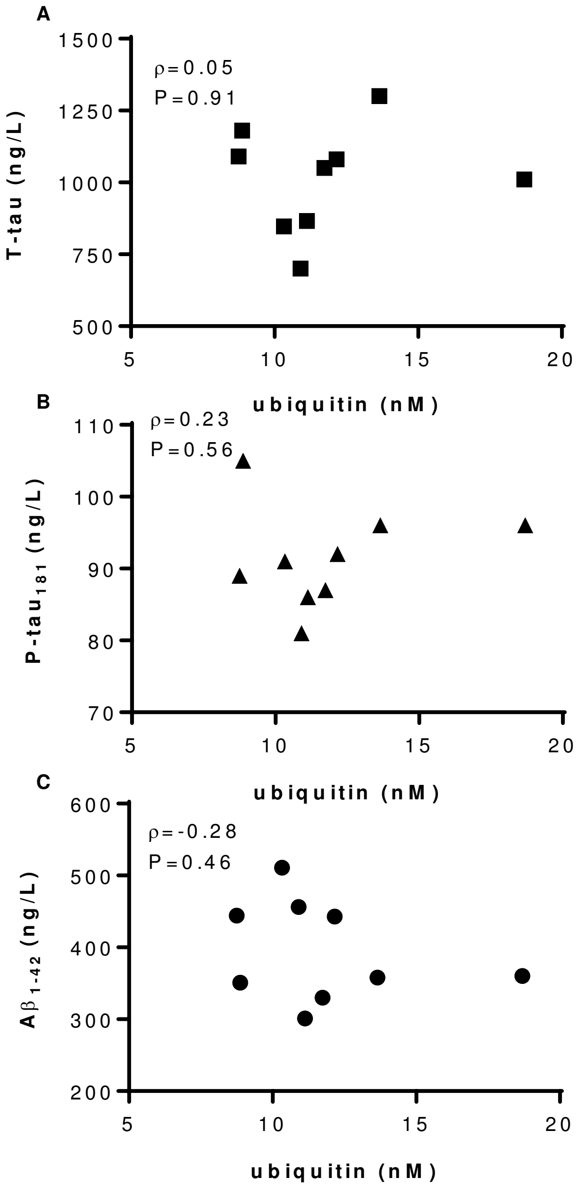

Figure S11. Correlations of Ubiquitin with CSF Core Biomarkers in Alzheimer's Disease in Study 1. Shown are scatter plots for the concentration of ubiquitin and A, total tau protein (T-tau); B, tau protein phosphorylated at Thr181 (P-tau_{181}) or; C, the 42 amino acid long amyloid β peptide ($A\beta_{1\text{-}42}$). Indicated are Spearman's ρ and the P-value. n=9.

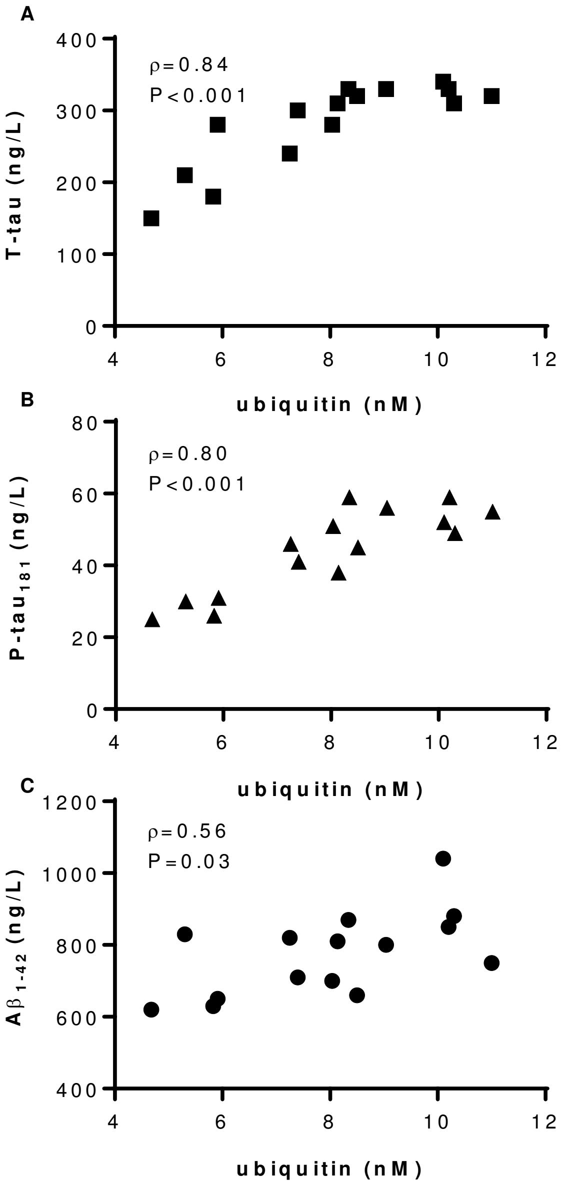

Figure S12. Correlations of Ubiquitin with CSF Core Biomarkers in Controls in Study 2. Shown are scatter plots for the concentration of ubiquitin and A, total tau protein (T-tau); B, tau protein phosphorylated at Thr181 (P-tau_{181}) or; C, the 42 amino acid long amyloid β peptide ($Aβ_{1-42}$). Indicated are Spearman's ρ and the P-value. n=15.

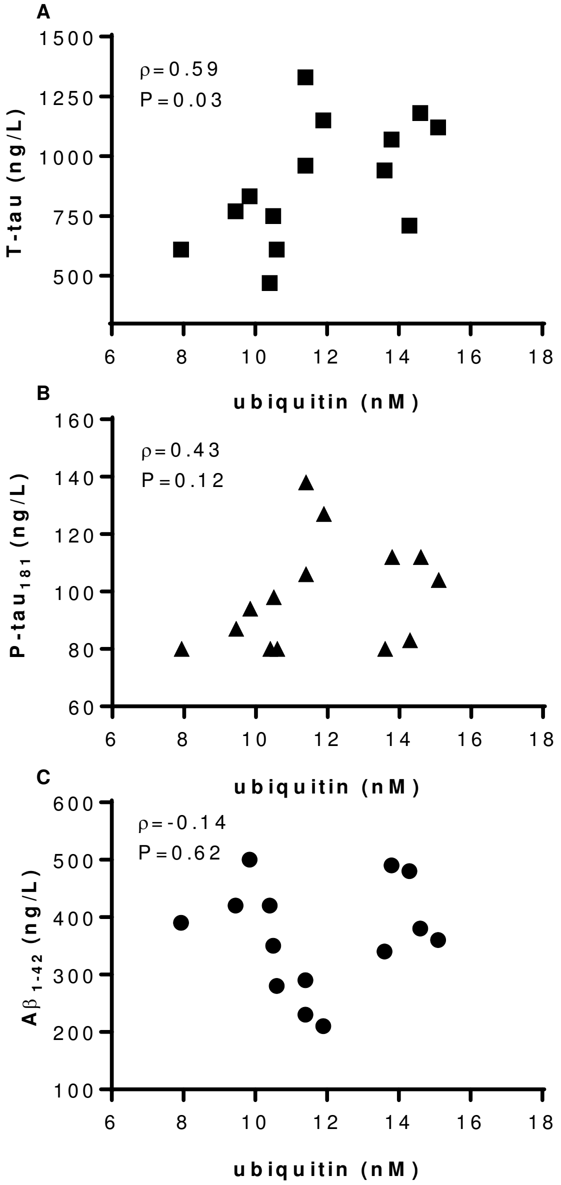

Figure S13. Correlations of Ubiquitin with CSF Core Biomarkers in Alzheimer's Disease in Study 2. Shown are scatter plots for the concentration of ubiquitin and A, total tau protein (T-tau); B, tau protein phosphorylated at Thr181 (P-tau$_{181}$) or; C, the 42 amino acid long amyloid β peptide (Aβ$_{1-42}$). Indicated are Spearman's ρ and the P-value. n=14.

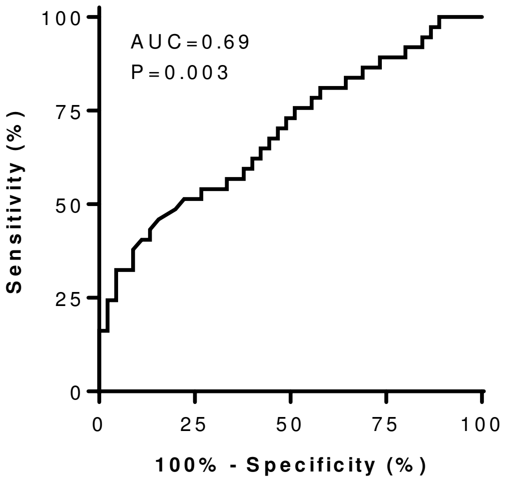

Figure S14. ROC Curve for Ubiquitin. Shown is a ROC curve for ubiquitin in participants with Alzheimer's disease (n=37) versus controls (n=45). Indicated are the calculated AUC and P-value.

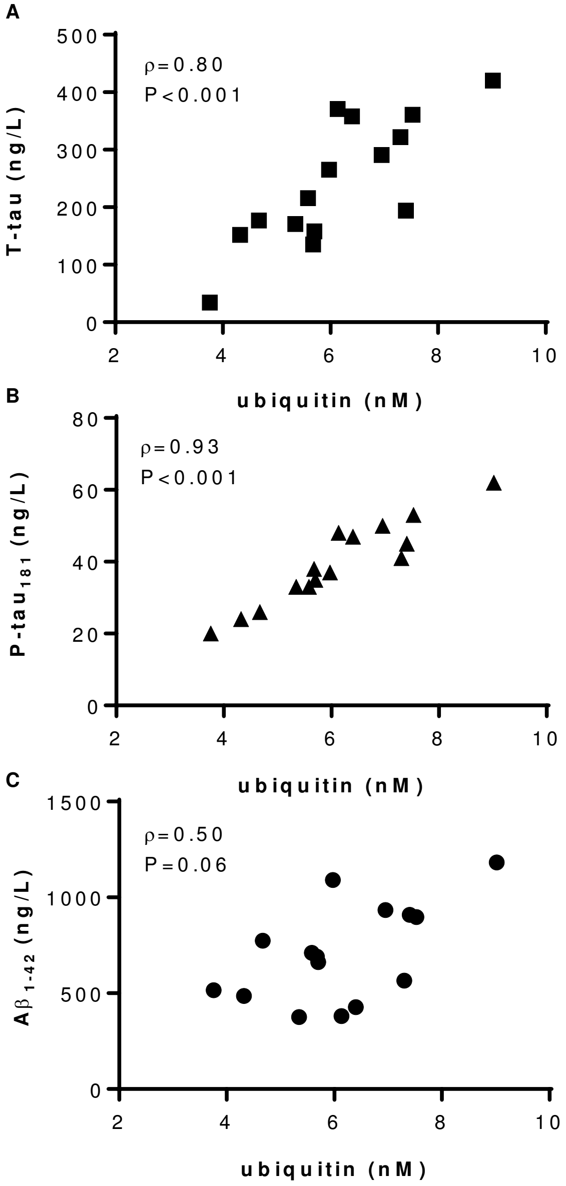

Figure S15. Correlations of Ubiquitin with CSF Core Biomarkers in Parkinson's Disease in Study 4. Shown are scatter plots for the concentration of ubiquitin and A, total tau protein (T-tau); B, tau protein phosphorylated at Thr181 (P-tau$_{181}$) or; C, the 42 amino acid long amyloid β peptide (A$\beta_{1\text{-}42}$). Indicated are Spearman's ρ and the P-value. n=15.

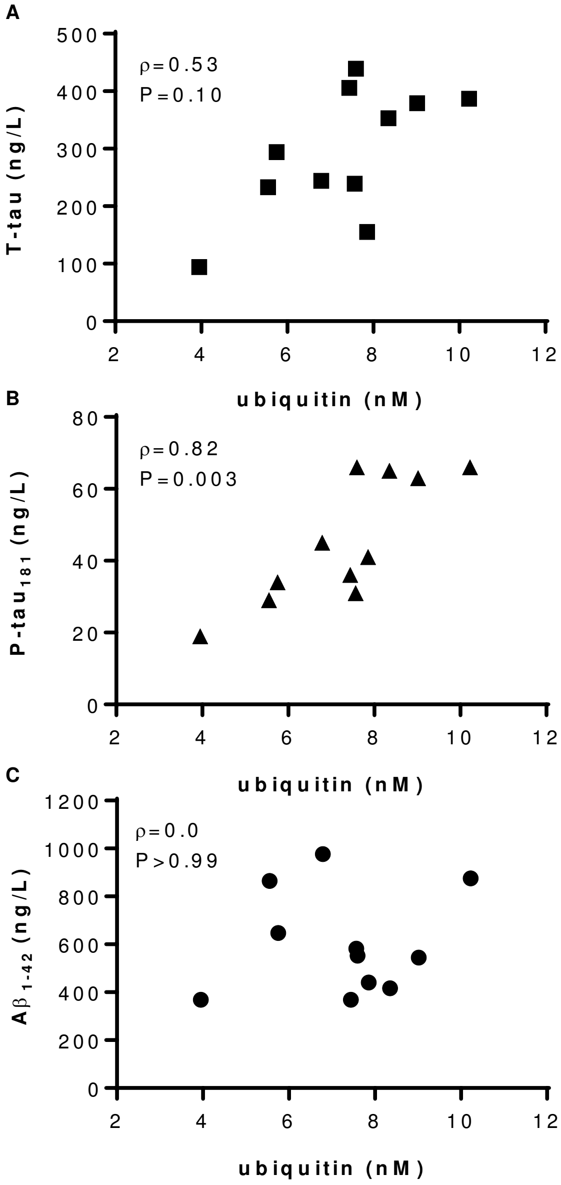

Figure S16. Correlations of Ubiquitin with CSF Core Biomarkers in Progressive Supranuclear Palsy in Study 4. Shown are scatter plots for the concentration of ubiquitin and A, total tau protein (T-tau); B, tau protein phosphorylated at Thr181 (P-tau_{181}) or; C, the 42 amino acid long amyloid β peptide (A$\beta_{1\text{-}42}$). Indicated are Spearman's ρ and the P-value. n=11.

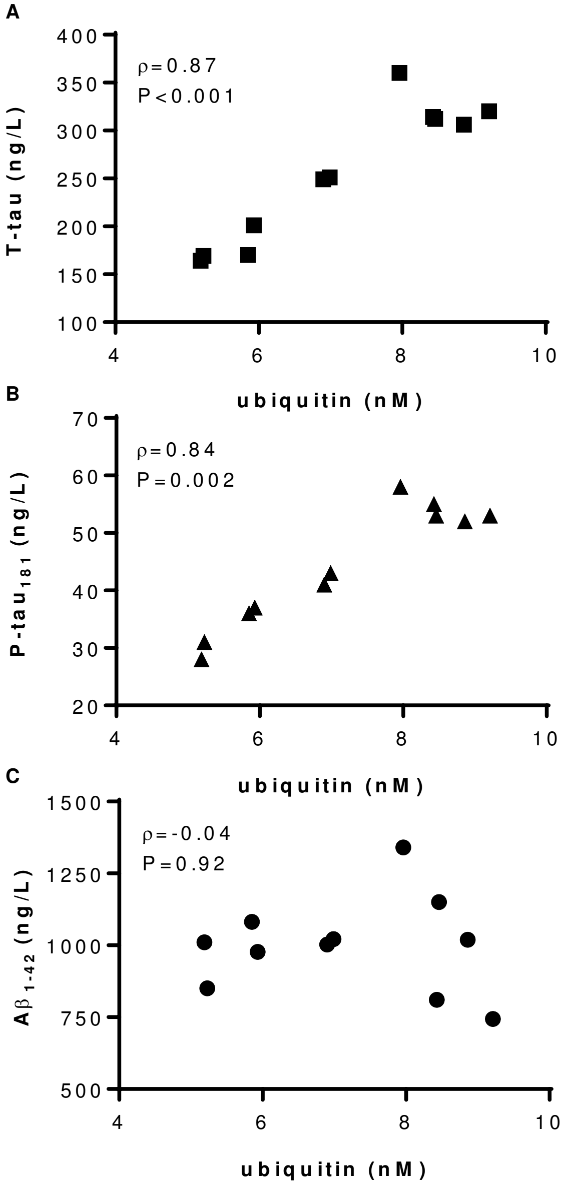

Figure S17. Correlations of Ubiquitin with CSF Core Biomarkers in Controls in Study 4. Shown are scatter plots for the concentration of ubiquitin and A, total tau protein (T-tau); B, tau protein phosphorylated at Thr181 (P-tau_{181}) or; C, the 42 amino acid long amyloid β peptide ($A\beta_{1\text{-}42}$). Indicated are Spearman's ρ and the P-value. n=11.
